# Should We Always Use Antibiotics after Urodynamic Studies in High-Risk Patients?

**DOI:** 10.1155/2018/1607425

**Published:** 2018-11-05

**Authors:** Pawel Miotla, Sara Wawrysiuk, Kurt Naber, Ewa Markut-Miotla, Pawel Skorupski, Katarzyna Skorupska, Tomasz Rechberger

**Affiliations:** ^1^2nd Department of Gynaecology, Medical University of Lublin, Lublin, Poland; ^2^Department of Urology, Technical University of Munich, Munich, Germany; ^3^Department of Paediatric Pulmonology and Rheumatology, Medical University of Lublin, Lublin, Poland

## Abstract

**Aim:**

The aim of this observational study was to evaluate the effectiveness of a phytotherapic drug (Canephron N) in preventing urinary tract infection (UTI) in high-risk women undergoing urodynamic studies (UDS).

**Methods:**

The study protocol was approved by the local institutional ethical committee. Adult women with at least one risk factor for acquiring UTI (defined as: age over 70, elevated postvoid residual urine>100 ml, recurrent UTI, pelvic organ prolapse (POP) ≥II in POP-Q scale, and neurogenic bladder) had received after UDS either a single oral dose of fosfomycin trometamol (FT) (3 grams) or a phytodrug containing centaury herb, lovage root, and rosemary leaves (5 ml taken orally three times daily for one week). All patients included in the study had no pyuria according to urine dipstick (nitrite and/or blood and/or leukocyte esterase) and negative urine culture (CFU < 10^3^/ml) before UDS. Urine samples were also tested 7 days after UDS.

**Results:**

Seventy-two high-risk participants completed the study. Seven days after urodynamic studies UTI symptoms, pyuria (nitrite and/or blood and/or leukocyte esterase) and bacteriuria with* E. coli *occurred in two patients (one (2.8%) in the FT and one (2.7%) in the phytodrug group, respectively). No statistical differences in UTI incidence were found between both treatment groups. We did not observe any additional adverse events in both groups. The major disadvantage of prophylaxis with the phytodrug as compared to FT was the necessity of continuing therapy for 7 days.

**Conclusion:**

Prophylaxis of UTI with a phytodrug (Canephron N) may be considered a good alternative to antibiotic prophylaxis use after UDS in high-risk female patients.

## 1. Introduction

Urodynamic studies (UDS) are especially used to evaluate lower urinary tract function in patients with bladder outlet obstruction, urinary incontinence, and neurogenic bladder dysfunction [1]. Urodynamic studies consist of series of tests, which can be helpful in proper recognition of abnormalities in the lower urinary tract. It has been already published that UDS, in many cases, significantly changed the ordering clinician's clinical impression of the patient's diagnosis for stress urinary incontinence and for urgency urinary incontinence [2]. UDS can also help in the better understanding of dysfunctions in patients with neurologic disorders, and in comprehending the changes in lower urinary tract functions in patients with pelvic gynaecologic cancer before and after radical treatment [3, 4]. UDS are an invasive procedure that involves catheterization; therefore, urinary tract infection (UTI) or bacteriuria may be observed after UDS, with an incidence of bacteriuria ranging from 1.5 to 30% [5].

The main value of prophylaxis is to decrease the risk of serious infection complications in patients after invasive procedures caused by the presence of bacteriuria. There is still no consensus on whether antibiotic prophylaxis is necessary for patients undergoing UDS. In the randomized study conducted on 270 patients published by Hirakauva* et al*., antibiotic prophylaxis before UDS did not reduce the incidence of UTI in women [5]. In the past, Cundiff* et al*. came to the same conclusion in their research. There was no statistically significant difference in bacteriuria between female patients receiving two doses of nitrofurantoin 100 mg and patients receiving placebo after combined urodynamics and cystourethroscopy [6]. On the other hand, Latthe* et al*., based on the results of randomized controlled studies, noticed a 40% reduction in the risk of significant bacteriuria with the administration of prophylactic antibiotics of different dose, type, and duration after UDS [7]. Therefore, it seems reasonable to reduce bacteriuria with antibiotic prophylaxis, because its rate correlates with the rate of infectious complications after invasive procedures [8].

Due to growing antibiotic resistance and weak evidence for routine antibiotic use in UDS prophylaxis, Tsai* et al*. investigated 261 patients and recommended that prophylactic antibiotics should be given only to high-risk patients [9]. Furthermore, Nadeem* et al*. also suggested giving antibiotics only to high-risk patients [10]. Unfortunately, it is still unclear which patients should be treated as high risk when it comes to UDS. Some potential risk factors are, however, considered in urogenital operations, including female gender [1], older age, diabetes mellitus, multipara (>3) [9], advanced organ prolapse, hypothyroidism, and body mass index >30 [11]. Although Cameron* et al*. did not recommend routine antibiotic prophylaxis in patients with diabetes mellitus, they did define risk factors for the development of UTI after UDS. These are neurogenic lower urinary tract dysfunction, elevated postvoid residual urine (PVR), asymptomatic bacteriuria, immunosuppression, age >70, and patients with an indwelling catheter [12]. Indeed, the use of prophylactic antibiotics is still controversial due to their many adverse effects and because of the increase of resistance of bacterial uropathogens. It is important, hence, to find a balance between the symptoms and risk associated with UTI, and with costs, adverse effects, and growing resistance to antibiotics [13].

Canephron N (Bionorica, Germany) is a phytotherapeutic drug with diuretic, spasmolytic, anti-inflammatory, antibacterial, and nephroprotective properties. The main ingredients of Canephron N are century herbs, lovage roots, and rosemary leaves. It is recommended for both adults and children. Moreover, it may also be used during pregnancy and breast feeding [14]. Because of Canephron's safety and positive impact on inflammatory processes within the urinary tract, we have decided to assess this phytodrug as a potential alternative to the use of antibiotics after UDS in high-risk patients.

The aim of this study was to evaluate the effectiveness of Canephron N in comparison to routine prophylaxis with fosfomycin trometamol (FT) (Zambon, Italy) in preventing UTIs in female patients undergoing urodynamic studies.

## 2. Materials and Methods

The protocol of this observational study was approved by the local institutional ethical committee. Urodynamic testing, including cystometry with bladder catheterisation, was conducted in women with mixed urinary incontinence, neurogenic bladder, or unclear lower urinary tract symptoms (LUTS). All participants were informed about the potential adverse events of FT and phytodrug and then gave written informed consent. In the study, women with at least one risk factor for acquiring UTI (defined as: age over 70, elevated postvoid residual urine (PVR)>100 ml, recurrent UTI, pelvic organ prolapse (POP) ≥II in POP-Q scale [15], and neurogenic bladder) received alternately after UDS, either a single oral dose of fosfomycin trometamol (3 grams) or a phytodrug (Canephron N) containing centaury herb, lovage root, and rosemary leaves (5 ml taken orally three times daily for one week). Simple randomization was used from pseudorandom numbers generated by a computer to allocate patients into the study groups in a ratio of 1:1.

All patients included in the study had no pyuria (nitrite and/or blood and/or leukocyte esterase) according to urine dipstick tests and negative urine culture (CFU < 10^3^/ml) before UDS. Standard aseptic procedure of catheterization during UDS was performed in all participants. Urine samples collected from clean-catch midstream were also tested with dipstick 7 days after UDS. All patients were also informed to contact the hospital immediately in case of any UTI symptoms if they occurred before scheduled visit.

The primary endpoint was the presence of UTI symptoms and/or positive dipstick for pyuria (nitrite and/or blood and/or leukocyte esterase) and/or bacteriuria (CFU>10^3^/ml) at follow-up visit. Secondary endpoint included the assessment of potential adverse events during the follow-up period.

Statistical analysis was performed with Statistica StatSoft, version 10 package, using the unpaired or paired t test and the *χ*2 test, as appropriate. A *p* value < 0.05 was considered statistically significant throughout.

## 3. Results 

To the best of our knowledge, our study is the first, which compares the efficacy of phytodrug (Canephron N) to fosfomycin trometamol (3g) in preventing bacteriuria or symptomatic UTI after UDS. Baseline demographic characteristics were similar between both groups (Table 1).

**Figure 1:**
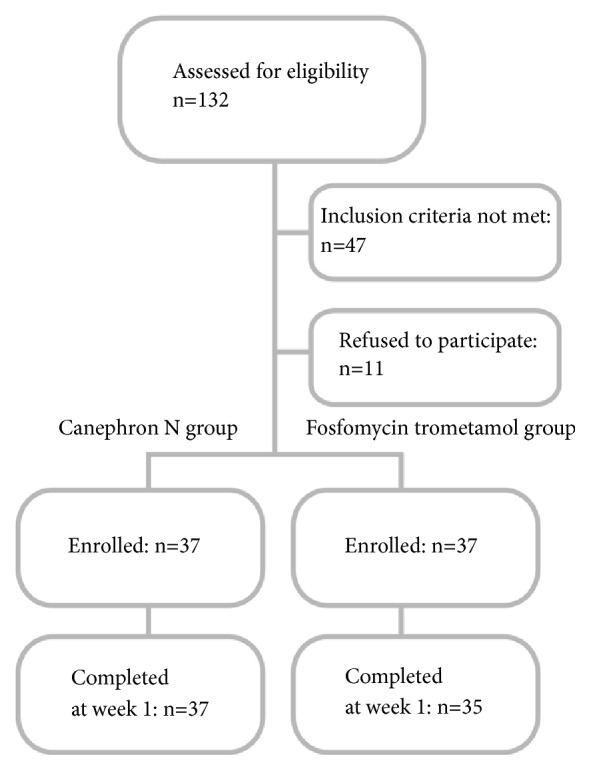
Flowchart of the participants in the study.

Seventy-two women completed treatment and the follow-up visit conducted at week 1 after UDS (see flowchart - Figure 1). Eleven patients with well-controlled diabetes mellitus type 2 were included in the study (5 in the FT and 6 in the phytodrug group, respectively). No statistical differences in incidence of diabetes mellitus were found between both treatment groups. In seven patients (3 in the FT and 4 in the phytodrug subgroup, respectively) menopausal hormone therapy (MHT), local or systemic, was continued during the study. There was no statistically significant difference in MHT administration between both groups.

In our study, seven days after urodynamic studies, UTI symptoms and pyuria (nitrite and/or blood and/or leukocyte esterase) according to urine dipstick tests occurred in two patients, one (2.8%) in the FT and one (2.7%) in the phytodrug group, respectively. The patient with urinary tract infection in the FT group had recurrent UTI in her medical history, whilst the participant in the phytodrug group presented POP-Q stage III in her gynaecological assessment, as well as increased PVR (180 ml). In both patients, urine culture was assessed, and* E. coli *(CFU/ml 10^6^) was recognized as a pathogen responsible for UTI development. We did not observe any additional adverse events in both groups. Indeed, no statistical differences in UTI incidence were found between both treatment groups; however 10 patients in phytodrug group reported the necessity of continuing therapy for 7 days as the major disadvantage of such prophylaxis.

## 4. Discussion

During urodynamic testing, there is a need to place a catheter into the bladder; therefore, it can result in UTI, and the incidence of UTI after urodynamic testing may vary from 1% to 30% [16–18]. The presence of UTI may also exacerbate urinary incontinence and other LUTS [19]. Hence, it is reasonable to find out if a patient truly requires antibiotic prophylaxis of UTI after UDS and to assess the efficacy of alternative pharmacotherapy options.

In the study published by Nóbrega* et al*., multivariate analysis with multiple logistic regression was conducted to assess the risk factors which are associated with bacteriuria and UTI after UDS. Based on the results collected from 232 women who underwent UDS, the authors found that body mass index (BMI) >30, advanced pelvic organ prolapse, and hypothyroidism are responsible for significant increase of risk bacteriuria, whilst only BMI >30 was associated with greater incidence of UTI after UDS [11].

Cameron* et al*., based on a literature review and the expert opinions, recommended antibiotic prophylaxis before urodynamic testing for patients with such medical conditions as known relevant neurogenic lower urinary tract dysfunction, increased PVR, asymptomatic bacteriuria, immunodeficiency, and age >70. Their recommendations also suggest antibiotic prophylaxis for patients with any indwelling catheter or who perform clean intermittent self-catheterization. As a first choice of antimicrobial agents before UDS in high-risk patients, they recommended a single dose of trimethoprim‐sulfamethoxazole; however, the choice of prophylactic antibiotic should also include local pathogen resistance patterns [12]. We built upon these studies and recommendations in our recognition of high-risk patients in our study. Our choice of antibiotic prophylaxis was also associated with knowledge of local resistance of* E. coli* to trimethoprim‐sulfamethoxazole (which exceeds 22%), and, therefore, in our region, this antibiotic should be avoided in prophylaxis [20].

Antimicrobial resistance epidemiology is still changing and so should empiric treatment implications. In the study conducted by Naber* et al*. during a three-year period (from 2003 till 2006) in 10 different countries, 4264 patients were analyzed in terms of epidemiology and antimicrobial susceptibility of uropathogens. The results revealed that the most common bacteria,* Escherichia coli,* had a prevalence of 76.7%, and it showed the* E. coli* susceptibility rate to methicillin of 95.8%, nitrofurantoin of 95.2%, and ciprofloxacin of 91.8%. The lowest rate was found for ampicillin (45.1%) [21]. In a similar study conducted by Miotla et al., also in a three-year period (from 2013 till 2015) 4453 patients were evaluated. Herein, the most common uropathogens cultured from urine samples were* E. coli* with a slightly lower prevalence of 65.5%. The resistance rate of* E. coli* strains for antibiotics mentioned earlier was slightly higher. Direct comparison between the ARESC study and our results shows mainly an increase of* E. coli* resistance to ciprofloxacin (10.7% and 22.7%, respectively, in the premenopausal and the postmenopausal groups) [22].

In UTI treatment, resistance rates should always be taken into consideration. For example, resistance of* E. coli* varies considerably within Europe; thus, ciprofloxacin is only recommended for empirical therapy when the resistance rate of* E. coli *is lower than 10-20% [22]. For these reasons (high resistance rates to trimethoprim‐sulfamethoxazole and fluoroquinolones), in our study, we chose fosfomycin trometamol (3g) as a prophylaxis in high-risk patients. Furthermore, the choice of phytodrug as a comparator was associated with the concerns of antimicrobial resistance, which is considered to be a major health threat. Our first dosage started after the intervention according to our local guidelines and previously published analyses considering antibiotic proxylaxis after UDS [23, 24].

Multidrug-resistant bacteria infections are associated not only with highest costs of treatment, but also with increased patient mortality and morbidity. Whether reduced antibiotic consumption can restore antibiotic susceptibility, Sundgvist* et al*. performed a very interesting intervention in which the use of trimethoprim was decreased by 85% due to voluntary restriction of its use in a certain area for 24 months. The results of the study were very promising, but the effect was rather disappointing. There was no statistically significant change in resistance of E. coli against trimethoprim. This study showed that, once bacterial resistance is established, it has a low possibility of reverting itself [25]. Nevertheless, it seems reasonable to encourage reduced use of antibiotics even if the only benefit would be slowing down the rate of increasing resistance [26]. Therefore, to ascertain the effect of avoidance of unnecessary antibiotic consumption, we decided to use a Canephron N as a comparator for this study.

Gürbüz* et al*. assessed the efficacy of ciprofloxacin in a single dose (500 mg) taken orally 1 hour before UDS (n=141) vs. a single dose of FT (3 g) taken approximately 12 hours before the procedure (n=137) and vs. no treatment group (n=133). Herein, a significant bacteriuria developed in 12 female patients during the first week after UDS. Broken down, the rate of detection was 6 (4.3%) participants from the fluoroquinolone groups, 3 patients (1.6%) from the FT group, and 3 (2.3%) women from the no-prophylaxis subgroup.* E. coli *was cultured in half the UTI cases. The authors concluded that previous urogenital surgeries and female gender were associated with statistically increased risk for bacteriuria after UDS; however, via multiple logistic regression analysis, only past urogenital surgeries were responsible for the presence of bacteriuria [1]. The incidence of UTI in our FT (2.8%) and phytodrug (2.7%) was similar to the results published in the abovementioned study.

Foon* et al*. conducted a review study of nine randomized controlled trials (RCTs), which included 973 patients between the ages of 18 and 82. Patients in their study received different types of antibiotics either 24 hours before or up to 72 hours after UDS. The authors observed (in 5 RCTs) less incidence of UTI in participants who received prophylactic antibiotics in comparison to no-treatment groups (20% vs. 28%, respectively), with no statistical significance of this finding. Moreover, adverse events (AEs) were reported only in 2 RCTs; however, the rate of AEs did not reach 1.5% of all participants. Based on their results, they calculated that, statistically, 13.4 women needed to receive antibiotic prophylaxis to prevent one case of bacteriuria. Therefore, one of the final conclusions of that meta-analysis was the statement that prophylactic antibiotics can reduce the risk of bacteriuria after UDS, whilst data considering reduction of symptomatic UTIs are limited [13]. Interestingly, not all of the patients included in that analysis fulfilled restricted criteria for recognition as a high-risk group, and, what is more, the incidence of UTI in both groups was much higher when compared to the results of our study, 2.8% and 2.7% in the FT and the Canephron N groups, respectively. In prophylactic treatment, nonantimicrobial preventative methods should be considered first, as antibiotic prevention is risky in terms of resistance [27]. Gürbüz et al. postulated that FT seems to be a first choice of prophylaxis in patients at higher risk of UTI development after urodynamic study [1]. The results of our study showed that the efficacy of Canephron N seems to be similar to FT in the prevention of UTI after UDS.

The major limitations of our study include the relatively small group of participants and lack of male patients. The strengths of the study include very restricted inclusion criteria used for the recognition of high-risk patients and the very good follow-up achieved.

## 5. Conclusions

Prophylaxis of UTI with Canephron N may be considered a good and safe alternative to antibiotic prophylaxis used after UDS in high-risk female patients. Moreover, the usage of a phytodrug might be helpful in decreasing antibiotic consumption, as well as in the prevention of growing antibiotic resistance.

## Figures and Tables

**Table 1:** Demographic characteristics of patient groups.

Variable	Prophylaxis with fosfomycin trometamol (n=35)	Prophylaxis with phytodrug (n=37)	*p*
Age (years)	62.7 ±11.2	63.8 ±10.8	NS
BMI (kg/m^2^)	30.1 ±3.8	30.2 ±4	NS
Parity	2.1 ±1.12	2.3 ±0.97	NS
Menopause	28 (80%)	31 (83.7%)	NS

Continuous variables are presented as the mean±SD; categorical variables are presented as number and %.

## Data Availability

The data used to support the findings of this study are available from the corresponding author upon request.
